# Histology of the Primary Tumor Correlates with False Positivity of Integrated 18F-FDG-PET/CT Lymph Node Staging in Resectable Lung Cancer Patients

**DOI:** 10.3390/diagnostics13111893

**Published:** 2023-05-29

**Authors:** Fuad Damirov, Mircea Gabriel Stoleriu, Farkhad Manapov, Karen Büsing, Julia Dorothea Michels, Gerhard Preissler, Rudolf A. Hatz, Peter Hohenberger, Eric D. Roessner

**Affiliations:** 1 Department of Thoracic Surgery, Ludwig Maximilian University of Munich, 81377 Munich, Germany; stoleriu@helmholtz-muenchen.de (M.G.S.); rudolf.hatz@med.uni-muenchen.de (R.A.H.); 2 Department of Surgery, Division of Surgical Oncology and Thoracic Surgery, University Hospital Mannheim, University of Heidelberg, 68167 Mannheim, Germany; peter.hohenberger@umm.de (P.H.); eric.roessner@unimedizin-mainz.de (E.D.R.); 3 Institute for Lung Biology and Disease, Comprehensive Pneumology Center (CPC), Member of the German Lung Research Center (DZL), Helmholtz Zentrum München, 81377 Munich, Germany; farkhad.manapov@med.uni-muenchen.de (F.M.); gerhard.preissler@rbk.de (G.P.); 4 Department of Radiation Oncology, Ludwig Maximilian University of Munich, 81377 Munich, Germany; 5 Clinic for Radiology and Nuclear Medicine, University Hospital Mannheim, University of Heidelberg, 68167 Mannheim, Germany; karen.buesing@medma.uni-heidelberg.de; 6 Department of Pulmonology and Critical Care, Thoraxklinik Heidelberg gGmbH, University of Heidelberg, 69126 Heidelberg, Germany; julia.michels@med.uni-heidelberg.de; 7 Translational Lung Research Center (TLRC), Member of the German Lung Research Center (DZL), University of Heidelberg, 69126 Heidelberg, Germany; 8 Department of Thoracic Surgery, Robert Bosch Hospital, Teaching Hospital of University Tübingen, 70376 Stuttgart, Germany; 9 Department of Thoracic Surgery, Center for Thoracic Diseases, University Medical Center of the Johannes Gutenberg University Mainz, 55131 Mainz, Germany

**Keywords:** lung cancer, 18F-FDG-PET/CT, lymph node staging, false-positive lymph nodes, diagnostic accuracy, decision tree

## Abstract

This study aimed to evaluate the diagnostic accuracy and false positivity rate of lymph node (LN) staging assessed by integrated 18F-fluorodeoxyglucose positron emission computed tomography (18F-FDG-PET/CT) in patients with operable lung cancer to the tumor histology. In total, 129 consecutive patients with non-small-cell lung cancer (NSCLC) undergoing anatomical lung resections were included. Preoperative LN staging was evaluated in the relationship to the histology of the resected specimens (group 1: lung adenocarcinoma/LUAD; group 2: squamous cell carcinoma/SQCA). Statistical analysis was performed by the Mann–Whitney U-test, the chi^2^ test, and binary logistic regression analysis. To establish an easy-to-use algorithm for the identification of LN false positivity, a decision tree including clinically meaningful parameters was generated. In total, 77 (59.7%) and 52 (40.3%) patients were included in the LUAD and SQCA groups, respectively. SQCA histology, non-G1 tumors, and tumor SUVmax > 12.65 were identified as independent predictors of LN false positivity in the preoperative staging. The corresponding ORs and their 95% CIs were 3.35 [1.10–10.22], *p* = 0.0339; 4.60 [1.06–19.94], *p* = 0.0412; and 2.76 [1.01–7.55], and *p* = 0.0483. The preoperative identification of false-positive LNs is an important aspect of the treatment regimen for patients with operable lung cancer; thus, these preliminary findings should be further evaluated in larger patient cohorts.

## 1. Introduction

Lymph node (LN) staging is a mandatory step in the diagnosis of non-small-cell lung cancer (NSCLC) [1]. Since mediastinal LN involvement is frequently associated with locally advanced tumor stages or even inoperable lung tumors [2], correct staging is a necessary tool for an appropriate multimodal therapy concept [3]. Current guidelines on the diagnosis and treatment of lung cancer include integrated 18F-fluorodeoxyglucose positron emission computed tomography scan (18F-FDG-PET/CT) as a routine staging approach in all lung cancer patients admitted for surgery or undergoing neoadjuvant therapy [4]. Its predictive role as a “digital biopsy” has been comprehensively proven [5].

Despite its accuracy (90%), sensitivity (78–85%), and specificity (87–92%) [6,7], integrated 18F-FDG-PET/CT has its limitations due to the frequent preoperative nodal upstaging in lung cancer patients [8,9]. Specifically, the occurrence of 18F-FDG-PET/CT false-positive LN metastasis was reported in up to 61% of NSCLC patients [9,10], due to concurrent infectious, inflammatory, or interstitial lung diseases [11,12]. These conditions subsequently lead to an inappropriate lung cancer diagnosis and potentially delayed or denied surgical treatment, with a negative impact on the survival of NSCLC patients with primarily resectable tumors [13]. Recent studies showed that NSCLC patients experiencing biopsy-proven N2 LN might benefit from upfront surgery as a first-line therapy, with similar clinical outcomes to those patients undergoing induction therapy followed by surgical resection [14]. In particular, patients with N2 LN presented an improved overall survival when compared to those patients experiencing combined hilar and mediastinal lymph node involvement (N1 + 2 LN) [15].

Despite extensive research on the usefulness of the integrated 18F-FDG-PET/CT in the NSCLC staging [5,16,17], only a few reports specifically addressed the clinical features of the LN metastasis (e.g., topography, number of involved N1/N2 stations) in relation to the histological subtype of the primary tumor. Previous studies separately described the predictors of LN metastasis in either lung adenocarcinoma [18,19,20] or squamous cell carcinoma [21], leaving a comparative analysis of N2 LN metastasis in both groups still insufficiently addressed.

For these reasons, the analysis of the clinical predictors associated with LN false positivity in NSCLC patients is of crucial importance to better stratify patients at risk and to improve diagnostic and treatment strategies.

Based on these considerations, the present study aimed to evaluate the predictive value of the tumor histology in the false positivity rate of LN staging assessed by integrated 18F-FDG-PET/CT, with potential diagnostic and therapeutic consequences in the context of multimodal cancer treatment.

## 2. Materials and Methods

### 2.1. Study Population

**Figure 1:**
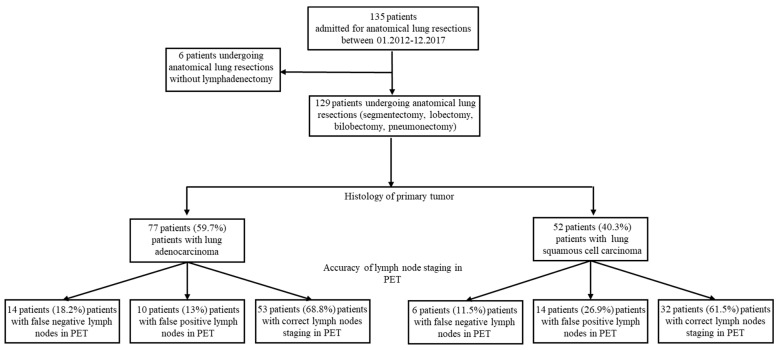
Study flow chart illustrating patient enrollment at study entry. Of 135 patients undergoing thoracic surgery, 6 (4.44%) patients underwent anatomical lung resections without lymphadenectomy. One hundred twenty-nine (95.7%) patients experiencing primary resectable malignant lung tumors were included. Based on the histology of the primary tumor, patients were categorized into two groups: lung adenocarcinoma group (LUAD, 77 patients, 59.7%) and squamous cell carcinoma group (SQCA, 52 patients, 40.3%). Based on the accuracy of 18F-FDG-PET/CT lymph node staging and the positivity of lymph nodes in the resected specimens, patients were categorized into three groups: false negative, false positive, and correct staging.

This single-center retrospective cohort study was conducted at the Division of Surgical Oncology and Thoracic Surgery of University Hospital Mannheim, Germany. All patients with resectable malignant primary lung tumors treated by anatomical resections (segmentectomy, lobectomy, bilobectomy, or pneumonectomy) between January 2012 and December 2017 were included. Patients undergoing anatomical lung resections without radical lymphadenectomy (*n* = 6) were excluded from the study. The local Ethical Committee’s approval was not required owing to the retrospective, observational, and anonymous nature of this study. Patient Consent Statements were not obtained due to the anonymous nature of the study. The study design is illustrated in Figure 1.

The analyzed outcome was the accuracy of the 18F-FDG-PET/CT lymph node staging to the reported intraoperative histological findings, by assessment of the false negatively, false positively, and correctly staged lymph nodes (LNs). The used definition is presented in Table 1.

18F-FDG-PET/CT scans were obtained using a 64-slice PET-CT scanner (Biograph Molecular CT system) based on 4 rings containing 48 detector blocks per ring. The used protocol was performed following the recommendations of the German Society of Radiology [22]. The standardized uptake value (SUV) was measured with a region-of-interest (ROI) technique and calculated by the software according to standard formulas.

Following the purpose of the study, the patient cohort was classified according to the histology of the resected specimens in patients with lung adenocarcinoma (LUAD group) and squamous cell carcinoma (SQCA group).

### 2.2. Data Assessments and Sources

Clinical data were collected from patient files of the Division of Surgical Oncology and Thoracic Surgery of University Hospital Mannheim, Germany. Lung tumor classification was performed following the 8th edition of the TNM staging system [23] and the World Health Organization (WHO) Histological Classification [24].

Clinical data encompass patients’ demographics (age, sex, cigarette smoke exposure, comorbidities), neoadjuvant therapy (chemotherapy, radiation therapy), preoperative lymph node staging investigations (18F-FDG-PET/CT, EBUS, mediastinoscopy), TNM and WHO histological classification of lung tumors, features of the surgical procedure (video-assisted thoracoscopic surgery (VATS), thoracotomy/open surgery), and postoperative 30-day mortality rate.

Following the 18F-FDG-PET/CT investigation, the size and maximum standardized uptake value (SUVmax) for the primary lung tumor and the lymph nodes were recorded. For the above-mentioned clinically relevant parametric variables, cut-off values were derived for clinical practicability.

### 2.3. Data Analysis

Parametric variables are presented as median values and quartiles [1st–3rd]. Binary categorical variables were analyzed by cross-tabulation using the chi-square test statistics or Fisher’s exact test. Continuous variables (age, tumor size, lymph node size, tumor SUVmax, lymph node SUVmax) were analyzed using the Mann–Whitney U-test. The binary logistic regression analysis was used to assess the independent value of the studied variables in predicting the accuracy of the preoperative 18F-FDG-PET/CT lymph node staging. The robustness of the independent predictors identified on multivariable analysis (enter method) was confirmed by forward and backward selection methods. The results were further validated by using the decision tree method obtained by two independent estimation approaches (exhaustive CHAID and CRT). Odds ratios (ORs) with 95% confidence intervals (CIs) were used to assess the prediction potential of the included variables. The cut-off values for the clinically relevant parameters were derived from receiver operator characteristics (ROC) analysis and the Youden method. *p*-values < 0.05 were considered statistically significant. The statistical analysis was performed using SPSS software (Version 26, IBM, Armonk, New York, USA).

## 3. Results

### 3.1. Study Population, Clinical Demographics, and Lung Cancer Histology

Of 135 patients who were admitted for lung cancer resections, 129 patients fulfilled the inclusion criteria (48 (37.2%) female patients, 81 (62.8%) male patients; median [quartiles] age of 66.0 [59.5; 72.0] years). Overall, 77 patients (59.7%) experienced a lung adenocarcinoma (LUAD group), and 55 patients (42.6%) had a squamous cell carcinoma (SQCA group) in the resected specimens. The selection process and groups of patients are illustrated in Figure 1. The clinical demographics of the patients categorized by histology of the primary tumor are presented in Table 2.

Female patients and well-differentiated tumors (G1) were significantly more frequent in the LUAD group (45.5% vs. 25.0%, *p* = 0.0184 and 16.9% vs. 1.9%, *p* = 0.0074). Specifically, smoker status, comorbidities, and neoadjuvant treatment, as well as tumor topography, size, and stage were not significantly associated with tumor histology (Table 2).

### 3.2. Surgical Approach and Lung Cancer Histology

Overall, 75 (58.1%) of the patients presented tumors on the right side and 54 (41.9%) on the left side. Of 129 patients, 77 (59.7%), 46 (35.7%), and 6 (4.7%) patients experienced lung tumors localized in the upper, lower, and middle lobes, respectively. Centrally located tumors were reported in 33 (25.6%) of the patients. In 80 (62%) patients, an open surgical approach was required. The characteristics of the surgical approaches are illustrated in Table 3.

The resection side and extent were not significantly correlated to the intraoperative histology. Open procedures were more frequent in the SQCA group (55.8% vs. 71.2%, 0.0789), whereas minimally invasive procedures were more frequent in the LUAD group (40.3% vs. 26.9%, *p* = 0.1190). Overall, five (3.8%) death events were reported in the whole cohort (two patients (2.6%) in the LUAD group and three patients (5.8%) in the SQCA group, *p* = 0.3599).

### 3.3. Integrated 18F-FDG-PET/CT and Lung Cancer Histology

Centrally located tumors were significantly more frequent in the SQCA group (18.2% vs. 36.5%, *p* = 0.0191). The frequency of tumors > 3 cm was almost similar in both groups (41.6% LUAD, 48.1% SQCA, *p* = 0.4646). Of note, a higher SUVmax of the primary tumor was significantly more frequent in the SQCA group (20.3% vs. 45.8%, *p* = 0.0027). Correct LN staging based on integrated 18F-FDG-PET/CT and intraoperative histology was reported in 68.8% of the cases in the LUAD group and 61.5% of the cases in the SQCA group. False-negative LNs were identified in 20 patients (15.5%), with no significant differences between LUAD and SQCA groups (18.2% vs. 11.5%, *p* = 0.3065).

False-positive LNs were reported in 24 out of 129 patients (18.6%). The LN false positivity was significantly higher in the SQCA group when compared to the LUAD group (26.9% vs. 13.0%, *p* = 0.0460). The radiological features of the tumors assessed by integrated 18F-FDG-PET/CT categorized by the histology of the primary tumor are summarized in Table 4.

### 3.4. Logistic Regression Analysis of Risk Factors

The clinically meaningful parameters significantly associated with the intraoperative histology in the univariate analyses (female sex, centrally located tumors, well-differentiated tumors/G1, and SUVmax > 12.65) were included in the multivariable analysis to further analyze their independent predictive value. Specifically, differentiation grade, female sex, and LN false positivity were identified as independent factors predicting intraoperative histology. These results were also confirmed by using two additional selection methods for the multivariable analyses (forward and backward LR), suggesting a certain grade of robustness (corresponding ORs and their 95% CIs were 11.39 (1.18–109,46), *p* = 0.0351; 3.80 (1.50–9.63), *p* = 0.0050; 3.12 (1.02–9.54), *p* = 0.0457, Table 5).

### 3.5. 18 F-FDG-PET/CT Lymph Node Staging Accuracy Stratified for Lung Cancer Histology

To assess the predictive values of the abovementioned clinically relevant parameters (Table 5) for the LN false positivity rate, a multivariable analysis was performed by incorporating the LN false positivity rate as a dependent variable. The following parameters were confirmed as significant predictors for LN false positivity (OR, 95% CI): squamous cell carcinoma histology (3.35 [1.10–10.22], *p* = 0.0339), non-G1 tumors (4.60 [1.06–19.94], *p* = 0.0412), and SUVmax tumor > 12.65 (2.76 [1.01–7.55], *p* = 0.0483). The results of the logistic regression analysis are presented in Table 6.

**Figure 2:**
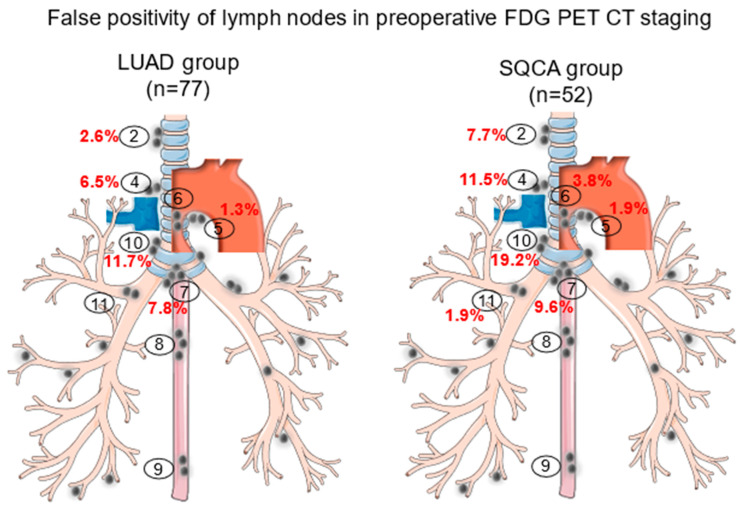
False positivity of lymph nodes in preoperative 18F-FDG-PET/CT staging in patients undergoing lung cancer resections classified by histology of the primary tumor. Encircled numbers (2 to 11) represent the sampled lymph node stations and red percentage figures the frequency of the false positivity of the respective lymph node stations.

The distribution of all sampled lymph node stations is presented in Table 7 and Figure 2.

**Figure 3:**
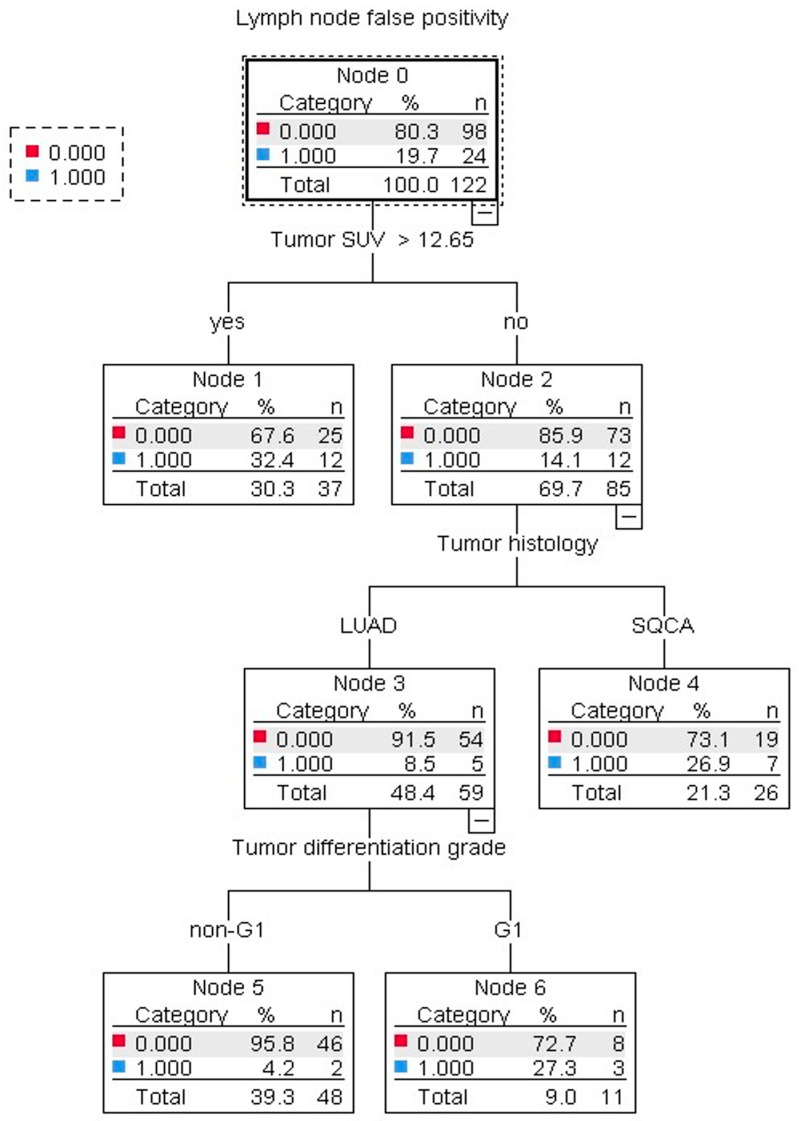
Decision tree illustration of the predictive factors for 18F-FDG-PET/CT lymph node false positivity in patients undergoing lung cancer resections. Tumor SUVmax > 12.65, squamous cell carcinoma histology, and G1 tumors in the LUAD group were associated with increased odds of lymph node false positivity. The analysis was performed by using the estimation procedure exhaustive CHAID (10-fold cross-validation) and confirmed by the CRT method with qualitatively unchanged results.

To design a clinically easy-to-use algorithm for the assessment of LN false positivity, a decision tree was generated (Figure 3) by using two estimation methods (exhaustive CHAID and CRT) with qualitatively unchanged results. By the multivariable analysis, an increased tumor SUVmax > 12.65, G1 tumors in the LUAD group, and SQCA histology were confirmed as predictive factors for LN false positivity (Table 5 and Table 6).

## 4. Discussion

Correct staging is essential for the multimodal treatment of patients with NSCLC experiencing mediastinal LN involvement. An integrated 18F-FDG-PET/CT is the standard non-invasive diagnostic tool in lung cancer staging [25]. Consequently, an accurate definition of LN involvement is the prerequisite for effective primary therapy and is associated with the long-term OS of the patients [26].

Despite its accuracy (90%), sensitivity (78–85%), and specificity (87–92%) [6,7] for the mediastinal LN staging in patients with NSCLC, 18F-FDG-PET/CT may be associated with false-positive LNs in up to 61% of patients [9]. Thus, the assessment of the risk factors associated with LN false positivity is an important topic.

Based on these considerations, the present study analyzed the diagnostic accuracy of the preoperative 18F-FDG-PET/CT-based LN staging with a focus on the nodal false-positive rate concerning the histological classification of the primary tumor.

Our study revealed female sex, tumor differentiation grade, and LN false positivity to significantly correlate with the intraoperative histology of the primary tumor.

Previous studies showed a significant correlation between sex, differentiation grade, and histology of the primary tumor [27,28,29]. However, a potential association between LN false positivity in the preoperative screening and histology of the primary lung tumor remains an insufficiently addressed topic [30].

To assess the relationship between LN false positivity and intraoperative histology, a multivariable analysis was performed by incorporating the clinically meaningful parameters found to be significant in the univariate analysis. Further, the predictive value of these parameters was confirmed by the decision tree validation method, suggesting a certain degree of robustness. Accordingly, squamous cell carcinoma histology, non-G1 tumors, and tumor SUVmax > 12.65 were identified to significantly impact the LN staging diagnostic accuracy.

Previous studies discussed the worse overall survival and disease-free survival rates in patients experiencing squamous cell carcinoma in comparison to those with adenocarcinoma [31,32], while other studies associated squamous cell carcinoma histology with a modest response to chemotherapy and immunotherapy [33,34]. In addition, Lee et al. and Ismail et al. showed an increased likelihood of false-positive LN in patients with squamous cell carcinoma in comparison to those with adenocarcinoma [12,35], indirectly reflecting the importance of LN staging accuracy.

Concerning the differentiation grade of the primary tumor and the preoperative LN status, controversial findings exist. Specifically, Lococo et al. found that intermediate-grade tumors were independently correlated with false-negative 18F-FDG-PET/CT results (OR, 2.78; *p* = 0.005) [29], while Li et al. found well-differentiated tumors as a risk factor for false-positive uptake in the preoperative staging [36]. Our results showed non-G1 tumors to be more frequently associated with squamous cell carcinoma histology and NSCLC patients with false-positive LN.

In addition, previous reports addressing the automated quantification of tumor SUVmax found that a tumor SUVmax ≥ 8.25 was associated with an increased rate of LN false negativity [36,37]. Our results identified a tumor SUVmax > 12.65 to be an independent predictor of false-positive LN metastasis in NSCLC patients in both univariate and multivariable analysis. In accordance with previous results of Kaseda et al. and Li et al., tumor size was not significantly associated with LN false positivity.

Although current guidelines recommend the confirmation of the mediastinal LN involvement before surgery by non-invasive and invasive approaches [38], a systematic intraoperative lymphadenectomy should be performed in all patients with resectable NSCLC. Consecutively, the likelihood of LN false positivity increases with the number of intraoperatively dissected LN, as reported by Dai et al. [39].

The present study has several limitations. First, the reduced number of patients with false-positive LNs might limit the interpretation of the results concerning the histology of the primary tumor. Thus, the reported results have to be interpreted with caution, given the lower sensitivity of the Mann–Whitney U-test and chi-square test, when dealing with imbalanced and small-numbered patient groups. However, our findings assessed by univariate analysis were confirmed by multivariable analysis and independently validated by two estimation methods using the decision tree approach, suggesting a certain degree of robustness. In addition, the presented results were in accordance with previous studies and thus might represent a valid reference for larger prospective studies that might strengthen the conclusions of this study.

Second, the retrospective nature of this study does not permit the analysis of further parameters (e.g., smoking status or pulmonary comorbidities associated with mediastinal LN enlargement) that could potentially provide further valuable explanations for the LN false positivity in patients with resectable NSCLC.

## 5. Conclusions

The false positivity of integrated 18F-FDG-PET/CT LN staging is a common phenomenon in the clinical setting. False-positive LNs were more frequently reported in the SQCA group, non-G1 tumors, and tumor SUVmax > 12.65. Preoperative identification of false-positive LN is an important aspect of the multimodal treatment regimen in patients with operable lung cancer; thus, these preliminary findings should be further evaluated in larger patient cohorts.

## Figures and Tables

**Table 1:** Accuracy parameters derived from 18F-FDG-PET/CT and intraoperative lymph node specimens in lung cancer patients undergoing anatomical resection.

Accuracy Parameters	18F-FDG-PET/CT	Intraoperative Histology
False positivity	+	−
False negativity	−	+
Correct lymph node staging	+	+

Abbreviations: 18F-FDG-PET/CT: 18F-fluorodeoxyglucose positron emission computed tomography.

**Table 2:** Demographics of patients undergoing surgical resection of primary lung tumors classified by histology of the primary tumor.

Patient Demographics at Study Entry	LUAD Group *n* = 77	SQCA Group *n* = 52	*p*-Value
Age (median, quartiles [1st; 3rd]) years >65 years	65.00 [58.0; 71.5] 38/77 (49.4%)	68.0 [62.0; 73.0] 30/52 (57.7%)	0.1350 0.3519
Sex (*n*, %) Female Male	35/77 (45.5%) 42/77 (54.5%)	13/52 (25.0%) 39/52 (75.0%)	0.0184
Smokers Never Ex-smokers Never or Ex-smokers Active	13/77 (16.9%) 23/77 (29.9 %) 36/77 (46.8 %) 41/77 (53.2 %)	3/52 (5.8%) 24/52 (46.2%) 27/52 (51.9%) 25/52 (48.1%)	0.0603 0.0594 0.5645 0.5645
Comorbidities (*n*, %) Respiratory Diabetes mellitus Other malignancies	24/77 (31.2%) 11/77 (14.3%) 29/77 (37.7%)	25/52 (48.1%) 10/52 (19.2%) 25/52 (48.1%)	0.0523 0.4555 0.2395
Previous treatments (*n*, %) Neoadjuvant chemotherapy Neoadjuvant radiation therapy	5/77 (6.5%) 0/77 (0.0%)	2/52 (3.8%) 1/52 (1.9%)	0.5150 0.2219
Lymph node staging investigations (*n*, %) EBUS Mediastinoscopy	20/77 (26.0%) 6/77 (7.8%)	21/52 (40.4%) 5/52 (9.6%)	0.0847 0.7161
Tumor side (*n*, %) Left Right	28/77 (36.4%) 49/77 (63.6%)	26/52 (50.0%) 26/52 (50.0%)	0.1236
Tumor localization (*n*, %) Left upper lobe Left lower lobe Right upper lobe middle lobe Right lower lobe	22/28 (78.6%) 6/28 (21.4%) 27/49 (55.1%) 4/49 (8.2%) 18/49 (36.7%)	17/26 (65.4%) 9/26 (34.6%) 11/26 (42.3%) 2/26 (7.7%) 13/26 (50.0%)	0.2797 0.2797 0.2915 0.9430 0.2669
Tumor size in resected specimens (median, quartiles [1st; 3rd]) cm >3 cm (*n*, %)	2.70 [1.80–4.30] 32/75 (42.7%)	2.95 [1.73–4.60] 24/48 (50.0%)	0.5859 0.4256
Tumor differentiation grade (WHO 2015, *n*, %) G1 (well differentiated/low grade) G2 (moderately differentiated/intermediate) G3 (poorly differentiated/high grade)	13/77 (16.9%) 34/77 (44.2%) 29/77 (37.7%)	1/52 (1.9%) 28/52 (53.8%) 23/52 (44.2%)	0.0074 0.2799 0.4556
TNM8 classification (*n*, %) T_0_ T_1_ T_2_ T_3_ T_4_ T_0–1/_T_2_–_4_ T_0–2/_T_3–4_ T_0–3/_T_4_ Lymph node involvement (*n*, %) N_0_ N_1_ N_2_ N_0–1/_N_2_ N_0/_*n*_+_	2/77 (2.6%) 41/77 (53.2%) 18/77 (23.4%) 10/77 (13.0%) 6/77 (7.8%) 43/77 (55.8%) 61/77 (79.2%) 71/77 (92.2%) 48/77 (62.3%) 16/77 (20.8%) 13/77 (16.9%) 64/77 (83.1%) 48/77 (62.3%)	0/52 (0.0%) 21/52 (40.4%) 18/52 (34.6%) 9/52 (17.3%) 4/52 (7.7%) 21/52 (40.4%) 39/52 (75.0%) 48/52 (92.3%) 39/52 (75.0%) 9/52 (17.3%) 4/52 (7.7%) 48/52 (92.3%) 39/52 (75.0%)	0.2415 0.1515 0.1627 0.4970 0.9834 0.0850 0.5732 0.9834 0.1322 0.6246 0.1301 0.1301 0.1322
UICC tumor stage classification (*n*, %) 0 I II III IV 0-I/II-IV 0-II/III-IV 0-III/IV	2/77 (2.6%) 30/77 (39.0%) 21/77 (27.3%) 20/77 (26.0%) 4/77 (5.2%) 32/77 (41.6%) 53/77 (68.8%) 73/77 (94.8%)	0/52 (0.0%) 26/52 (50.0%) 18/52 (34.6%) 7/52 (13.5%) 1/52 (1.9%) 26/52 (50.0%) 44/52 (84.6%) 51/52 (98.1%)	0.2415 0.2147 0.3731 0.0867 0.3450 0.3444 0.0417 0.3450
Pleura invasion in histological specimens (*n*, %) yes no	8/77 (10.4%) 69/77 (89.6%)	6/52 (11.5%) 46/52 (88.5%)	0.837

Abbreviations: LUAD: lung adenocarcinoma; SQCA: squamous cell carcinoma; EBUS: endobronchial ultrasound; WHO: World Health Organization; TNM: tumor lymph node metastasis staging system; UICC: Union for International Cancer Control.

**Table 3:** Technical aspects of the tumor resection in primary lung cancer patients grouped by histology of the primary tumor.

Features of the Surgical Approach	LUAD Group *n* = 77	SQCA Group *n* = 52	*p*-Value
Resection side (*n*, %) Left Right	28/77 (36.4%) 49/77 (63.6%)	26/52 (50.0%) 26/52 (50.0%)	0.1236
Surgical approach (*n*, %) Open (thoracotomy) Minimally invasive (VATS)	43/77 (55.8%) 34/77 (44.2%)	37/52 (71.2%) 15/52 (28.8%)	0.0789 0.0789
Resection extent (*n*, %) Segmentectomy Lobectomy Bilobectomy Pneumonectomy	10/77 (13%) 61/77 (79.2%) 5/77 (6.5%) 1/77 (1.3%)	10/52 (19.2%) 36/52 (69.2%) 3/52 (5.8%) 3/52 (5.8%)	0.6506 0.1975 0.8671 0.1508
Mortality rate (*n*, %) 30-day	2/77 (2.6%)	3/52 (5.8%)	0.3599

Abbreviations: LUAD: lung adenocarcinoma; SQCA: squamous cell carcinoma; VATS: video-assisted thoracoscopic surgery.

**Table 4:** Morphologic features of the tumors assessed by 18F-FDG-PET/CT in primary lung cancer patients grouped by histology of the primary tumor.

Features of the Surgical Approach	LUAD Group *n* = 77	SQCA Group *n* = 52	*p*-Value
Tumor side (*n*, %) Left Right	28/77 (36.4%) 49/77 (63.6%)	26/52 (50.0%) 26/52 (50.0%)	0.1236
Tumor localization (*n*, %) Left upper lobe Left lower lobe Right upper lobe Middle lobe Right lower lobe	22/28 (78.6%) 6/28 (21.4%) 27/49 (55.1%) 4/49 (8.2%) 18/49 (36.7%)	17/26 (65.4%) 9/26 (34.6%) 11/26 (42.3%) 2/26 (7.7%) 13/26 (50.0%)	0.2797 0.2797 0.2915 0.9430 0.2669
Tumor topography (*n*, %) Central Peripheral	14/77 (18.2%) 63/77 (81.8%)	19/52 (36.5%) 33/52 (63.5%)	0.0191
Tumor size (median, quartiles [1st; 3rd]) cm >3 cm (*n*, %)	2.70 [1.90–3.75] 32/77 (41.6%)	2.95 [2.10–4.55] 25/52 (48.1%)	0.3097 0.4646
Lymph node size (median, quartiles [1st; 3rd]) cm >3 mm (*n*, %)	0.0 [0.0–1.05] 0/72 (0.0%)	0.00 [0.0–1.15] 1/44 (2.3%)	0.7446 0.1989
SUVmax of the primary tumor (median, quartiles [1st; 3rd]) cm >12.65 (*n*, %)	7.85 [4.48–11.53] 15/74 (20.3%)	12.0 [5.78–14.6] 22/48 (45.8%)	0.0078 0.0027
SUVmax of the positive lymph node (median, quartiles [1st; 3rd]) cm >1.25 (*n*, %)	0.0 [0.0–3.15] 25/77 (32.5%)	0.0 [0.0–3.45] 20/52 (38.5%)	0.5441 0.4835
Accuracy of lymph node staging: 18F-FDG-PET/CT ←→ histological specimen (*n*, %) False negative False positive Correct staging	14/77 (18.2%) 10/77 (13.0%) 53/77 (68.8%)	6/52 (11.5%) 14/52 (26.9%) 32/52 (61.5%)	0.3065 0.0460 0.3914
Time 18F-FDG-PET/CT ←→ surgery (median, quartiles [1st; 3rd]) days >30 days (*n*, %)	41 [24.0–65.5] 49/77 (63.6%)	42 [27.0–63.0] 36/52 (69.2%)	0.7422 0.5109

Abbreviations: LUAD: lung adenocarcinoma; SQCA: squamous cell carcinoma; SUV: standardized uptake value; 18F-FDG-PET/CT: 18F-fluorodeoxyglucose positron emission computed tomography.

**Table 5:** Binary logistic regression model predicting the intraoperative histology in primary lung cancer patients undergoing major surgical resections.

Covariates Predicting Intraoperative Histology	Exp(B) [95% CI]	*p*-Value
Non-G1	11.39 [1.19–109.46]	0.0351
Female sex	3.80 [1.50–9.63]	0.0050
Lymph node false positivity	3.12 [1.02–9.54]	0.0457
SUVmax tumor > 12.65	2.40 [0.96–5.97]	0.0607

Abbreviations: Exp(B) = odds ratio, 95% confidence interval [lower bound–upper bound].

**Table 6:** Binary logistic regression model predicting false positivity of the integrated 18F-FDG-PET/CT-based lymph node staging in primary lung cancer patients undergoing major surgical resections.

Covariates for False Positive Lymph Node Staging	Exp(B) [95% CI]	*p*-Value
Squamous cell carcinoma histology	3.35 [1.10–10.22]	0.0339
Non-G1	4.60 [1.06–19.94]	0.0412
SUVmax tumor > 12.65	2.76 [1.01–7.55]	0.0483
Female sex	1.32 [0.47–3.75]	0.5971

Abbreviations: Exp(B) = odds ratio, 95% confidence interval [lower bound–upper bound].

**Table 7:** Accuracy of 18F-FDG-PET/CT-based lymph node staging for all lymph node stations, classified by histology of the primary tumor.

Lymph Node Station	18F-FDG-PET/CT Lymph Node Staging Accuracy LUAD Group, *n* = 77			18F-FDG-PET/CT Lymph Node Staging Accuracy SQCA Group, *n* = 52		
	False Positive	False Negative	Correct Staging	False Positive	False Negative	Correct Staging
2	2.6% (2/77)	3.9% (3/77)	2.6% (2/77)	7.7% (4/52)		
3			1.3% (2/77)			
4	6.5% (5/77)	2.6% (2/77)	2.6% (2/77)	11.5% (6/52)		
5	1.3% (1/77)		1.3% (1/77)	1.9% (1/52)	1.9% (1/52)	
6			1.3% (1/77)	3.8% (2/52)		
7	7.8% (6/77)	1.3% (1/77)	2.6% (2/77)	9.6% (5/52)	1.9% (1/52)	
8		1.3% (1/77)			1.9% (1/52)	
9		1.3% (1/77)			0% (0/52)	
10	11.7% (9/77)	6.5% (5/77)	3.9% (3/77)	19.2% (10/52)	1.9% (1/52)	3.8% (2/52)
11		10.4% (8/77)	3.9% (3/77)	1.9% (1/52)	7.7% (4/52)	5.8% (3/52)
12		1.3% (1/77)			0% (0/52)	1.9% (1/52)
13		1.3% (1/77)			0% (0/52)	

Abbreviations: LUAD: lung adenocarcinoma; SQCA: squamous cell carcinoma; 18F-FDG-PET/CT: 18F-fluorodeoxyglucose positron emission computed tomography.

## Data Availability

The datasets used and/or analyzed during the current study are available from the corresponding author on reasonable request.
